# Dynamic Protonation States Underlie Carbene Formation in ThDP-Dependent Enzymes: A Theoretical Study

**DOI:** 10.1021/acs.jpcb.3c03137

**Published:** 2023-09-25

**Authors:** Jon Uranga, Fabian Rabe von Pappenheim, Kai Tittmann, Ricardo A. Mata

**Affiliations:** † Institute of Physical Chemistry, Georg-August Universität Göttingen, Tammannstraße 6, 37077 Göttingen, Germany; ‡ Department of Molecular Enzymology, Göttingen Center of Molecular Biosciences, Georg-August Universität Göttingen, Julia-Lermonotowa-Weg 3, D-37077 Göttingen, Germany; § Department of Physical Biochemistry, Max-Planck-Institute for Multidisciplinary Natural Sciences, Am Faßberg 11, D-37077 Göttingen, Germany

## Abstract

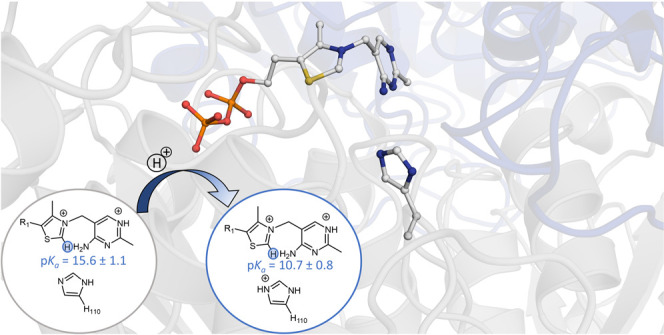

The activation mechanism of thiamine diphosphate (ThDP) in enzymes has long been the subject of intense research and controversial discussion. Particularly contentious is the formation of a carbene intermediate, the first one observed in an enzyme. For the formation of the carbene to take place, both intramolecular and intermolecular proton transfer pathways have been proposed. However, the physiologically relevant pH of ThDP-dependent enzymes around neutrality does not seem to be suitable for the formation of such reactive chemical species. Herein, we investigate the general mechanism of activation of the ThDP cofactor in human transketolase (TKT), by means of electronic structure methods. We show that in the case of the human TKT, the carbene species is accessible through a p*K*_a_ shift induced by the electrostatics of a neighboring histidine residue (H110), whose protonation state change modulates the p*K*_a_ of ThDP and suppresses the latter by more than 6 pH units. Our findings highlight that ThDP enzymes activate the cofactor beyond simple geometric constraints and the canonical glutamate. Such observations in nature can pave the way for the design of biomimetic carbene catalysts and the engineering of tailored enzymatic carbenes.

## Introduction

**Figure 1:**
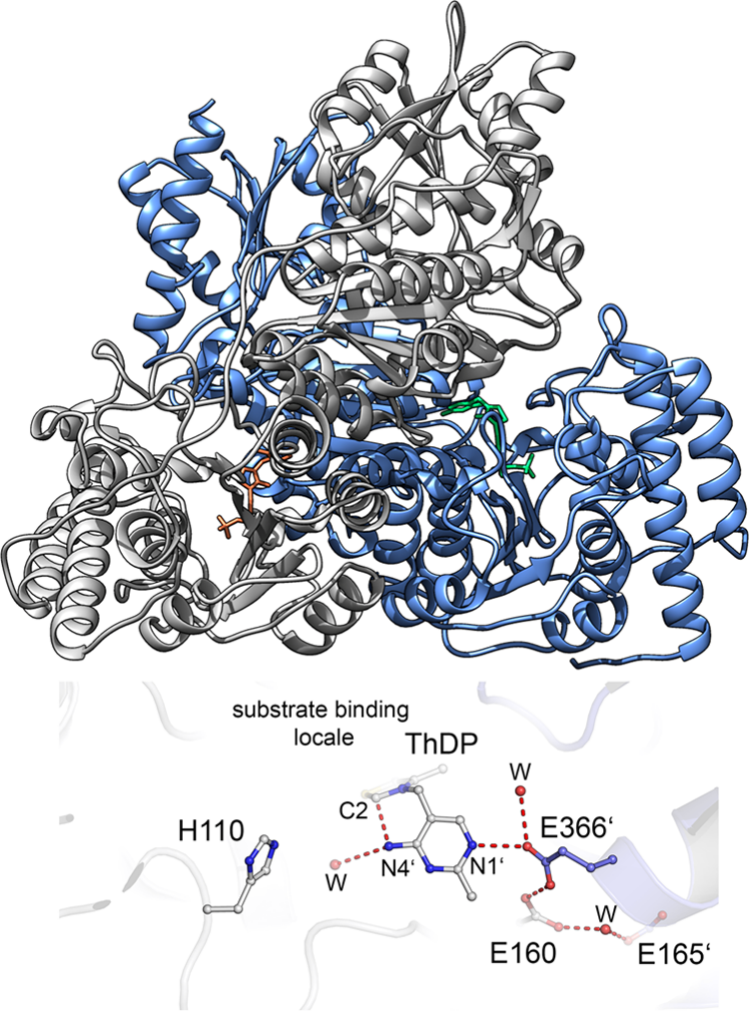
Top: TKT homodimer (PDB code 4KXW), with bound ThDP substrate in orange and green, highlighting the two active sites. Bottom: Representation of the active pocket in human transketolase, highlighting the ThDP cofactor and the nearby residues, which are used as a model in the cluster calculations.

**Figure 2:**
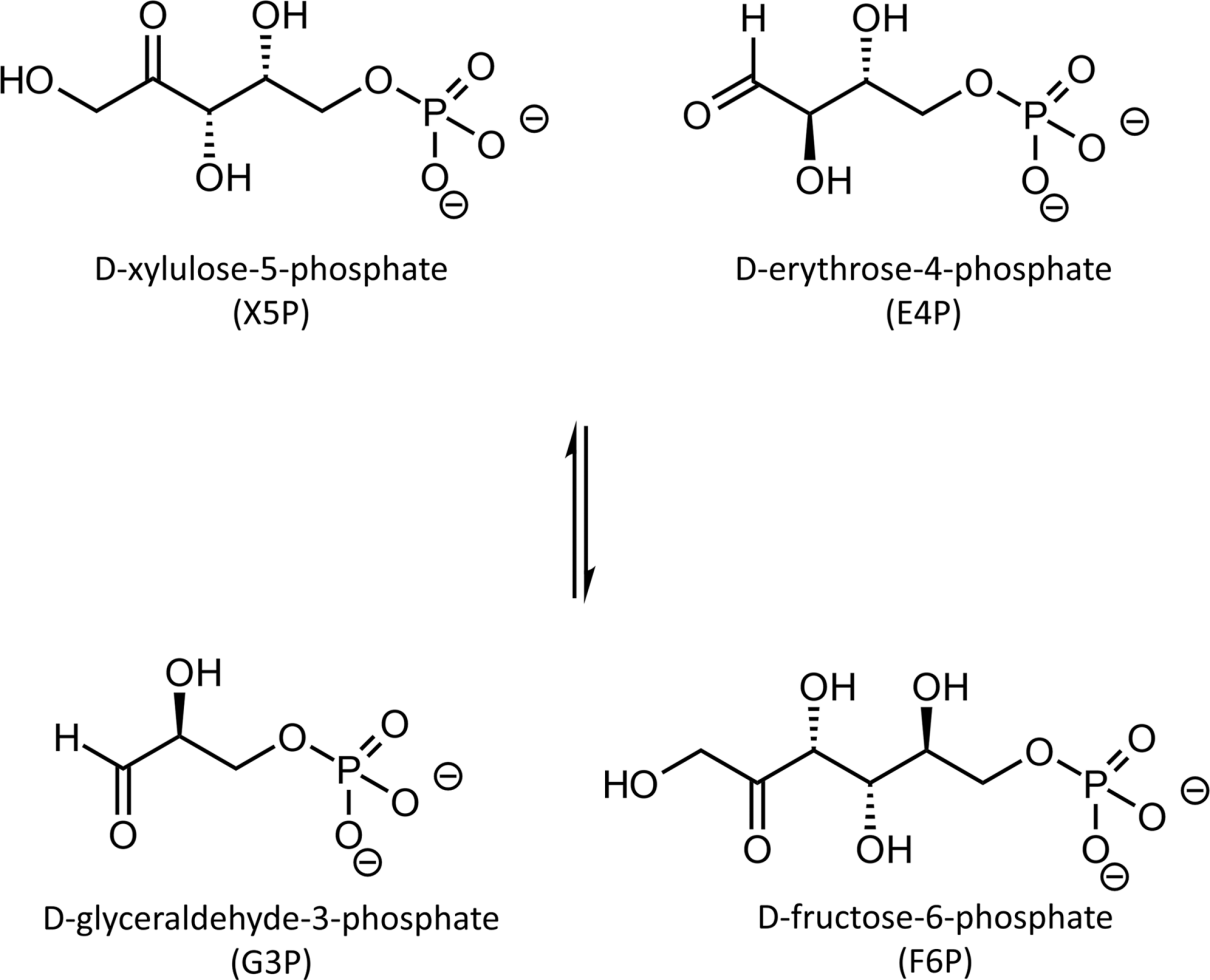
Carbon atom transfer reaction performed by TKT.

**Figure 3:**
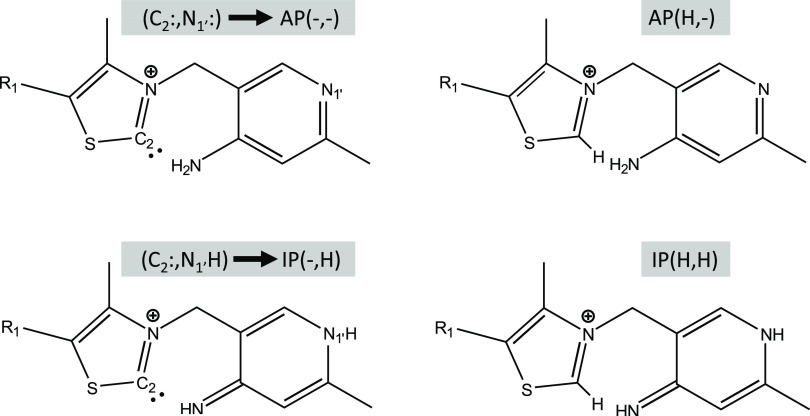
Lewis structure of the imino (IP) and amino (AP) ThDP tautomers, presenting the nomenclature that will be employed throughout the work. Two examples of the carbene are provided in gray (left), showing the nomenclature. In blue, two noncarbene tautomers are presented.

Enzymes are sophisticated machines that perform chemical reactions at rates and specificity greatly outmatching our current synthetic capabilities. Understanding this catalytic efficiency has stimulated lively scientific discussion and promoted different catalysis strategies (biomimetics). In other cases, one has come to realize that revolutionary tactics used in the lab were long under use in nature. The transketolase (TKT) enzyme is an extensively studied example of a thiamine diphosphate (ThDP)-dependent enzyme (see Figure 1), catalyzing the transfer of a two-carbon ketol group from a ketose donor to an aldose acceptor (X5P) (see Figure 2).^1^ The free energy barrier (Δ*G*^‡^), taking the kinetic constants and a simple Arrhenius expression, is documented to be around 17.5 kcal/mol.^2^ ThDP is a B1 vitamin-derived cofactor, whose reactivity is dominated by the activation of carbon C_2_ (see Figure 3), upon the loss of a proton. However, the exact nature of the resulting species is widely contested.

Meyer and co-workers first postulated and presented data in support of carbene formation in the pyruvate oxidase (POX) system, showing that the cofactor is in a tautomeric equilibrium and highlighting the presence of a water molecule close to the C_2_ atom.^3^ The carbene species is said to be characterized by a shortened angle S_1_–C_2_–N_3_ of 107.6°. Such chemical equilibria are not unique, and it has also been shown that different tautomers of the cofactor can be identified.^4,5^

The catalytic mechanism of human transketolase, including cofactor activation, has already been addressed by several theoretical studies. Here, we address a subset of these results. In the reaction mechanism proposed by Prejanò et al.,^6^ the starting structure is the carbene form of ThDP. This activation process has already been mentioned to be energetically demanding, and it is not obvious to select such a state as a starting structure. In this vein, the catalytic action of human TKT remains unclear. Recently, some of the same authors have focused on an experimentally observed distortion of the cofactor, examining the possibility of intermediate destabilization.^7^ However, the obtained results show that the latter distortion does not have a major impact on the energetics of the catalyzed reaction.

On the activation mechanism itself, previous theoretical investigations focused on the intramolecular activation, that is the iminopyrimidine form of the cofactor acting as a base and directly abstracting the proton from the C_2_ position of the thiazolium moiety, thereby forming the aminopyrimidine form of the ThDP carbene.^8^ The study mentioned employs explicit water molecules and a dielectric continuum in order to represent the protein environment. In the same sense, Medina and Prejanò have recently studied the intramolecular activation mechanism of ThDP within the human TKT.^9^ However, it is unexpected to observe an exergonic process for the formation of such an unstable intermediate. Moreover, this mechanism directly conflicts with a previously suggested intermolecular activation pathway, by which a proton is transferred from the ThDP to a neighboring histidine residue.^10^ Indeed, it has previously been highlighted the importance of the histidine residues in the vicinity of the active site, H110 (in the human system) having been identified as a key player in the catalytic mechanism of ThDP.^11^ Thereby, it would be counterintuitive to find a mechanism in which the histidines do not have an active role.

In the proposed intermolecular path, a regular acid–base reaction takes place between the cofactor and the neighboring residue. One can formulate an equilibrium of two species A and B, which exchange a proton:
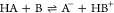
1Therefore, if the p*K*_a_ value of one of them is known, then one can obtain the p*K*_a_ value for the other species, employing a square scheme. In this sense, the usual p*K*_a_ value of a histidine is around 7,^12^ and using the mentioned strategy, one obtains a p*K*_a_ value of about 4 for the formation of the carbene in the ThDP, which is solely attributed to the mesomeric effect.^10^ To the best of our knowledge, such value has never been reported before; indeed, the usual p*K*_a_ values of thiamine are said to be greater than 14,^13^ and specifically a value of 18 was assigned to the carbene formation of free thiamine.^14^ p*K*_a_ shifts have previously been reported but such a shift would be without precedent.^15^ Finally, the obtained exergonic value for the formation of the carbene seems to be questionable and counterintuitive.

On the relative stability of the carbene species, structural theoretical studies have shown that carbene species can form up to two hydrogen bonds, by which they are thermodynamically stabilized.^16^ Such strong hydrogen bonds have been reported to be up to 20 kcal/mol, which may contribute to the lowering of the p*K*_a_ value.^13^

Among other reports, Hsu et al. proposed that the activation takes place through non-Kekulé diradicals based on crystal structure data.^17^ However, the structures obtained are in stark contrast to the data from other groups. Furthermore, model calculations carried out in the cofactor unequivocally show that the triplet/singlet biradical states are way too high in energy, as should be expected. Further details are later discussed in this manuscript.

**Figure 4:**
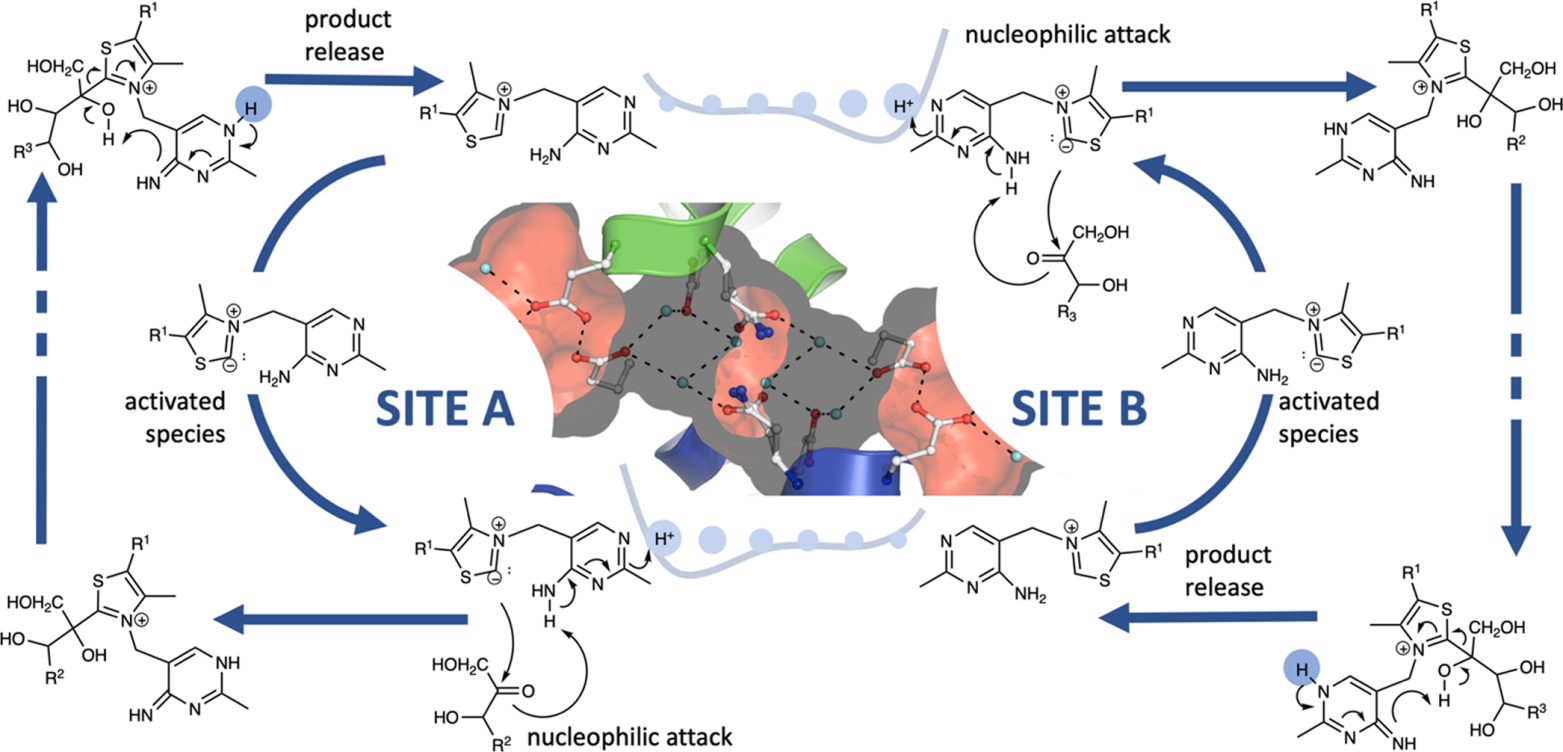
Scheme depicting the proposed allosteric mechanism in TKT, in relation to the different reaction steps. The carbene formation would take place with ThDP in its amino tautomeric form. Upon further reaction with the substrate, the proton balance would shift the potential between the sites, favoring the amino form in the other active pocket. From here onward, one obtains a cycle with successive substrate binding events, synchronizing the proton uptakes and respective activation of the cofactor.

The objective of the present study is to understand the formation of the carbene species, in particular for the human TKT system, while conducting a comprehensive assessment of the protonation states that have been shown to be of paramount importance.^18^ The carbene formation in ThDP is associated with the V-conformation adopted by the cofactor in the active site and the protonation state change promoted at the N_1′_ position through the canonical glutamate residue. The formation of carbene is strongly linked to the amino-imino tautomeric equilibrium, which in turn promotes an allosteric effect between the two active sites, as proposed.^19^ A scheme is provided in Figure 4, highlighting how the changes in the tautomer form at each site can be coupled to the reaction cycle.

The formation of a carbene is linked to a high p*K*_a_, value while the optimal pH of the TKT is reported to be in a range of 7.5–8.6.^20^ Hence, the formation of such an unstable chemical species is not straightforward, and the contradiction needs to be settled. The current work is focused on studying this activation step analyzing the p*K*_a_ shifts induced by the neighboring residues.

## Methods

### QM Calculations

**Figure 5:**
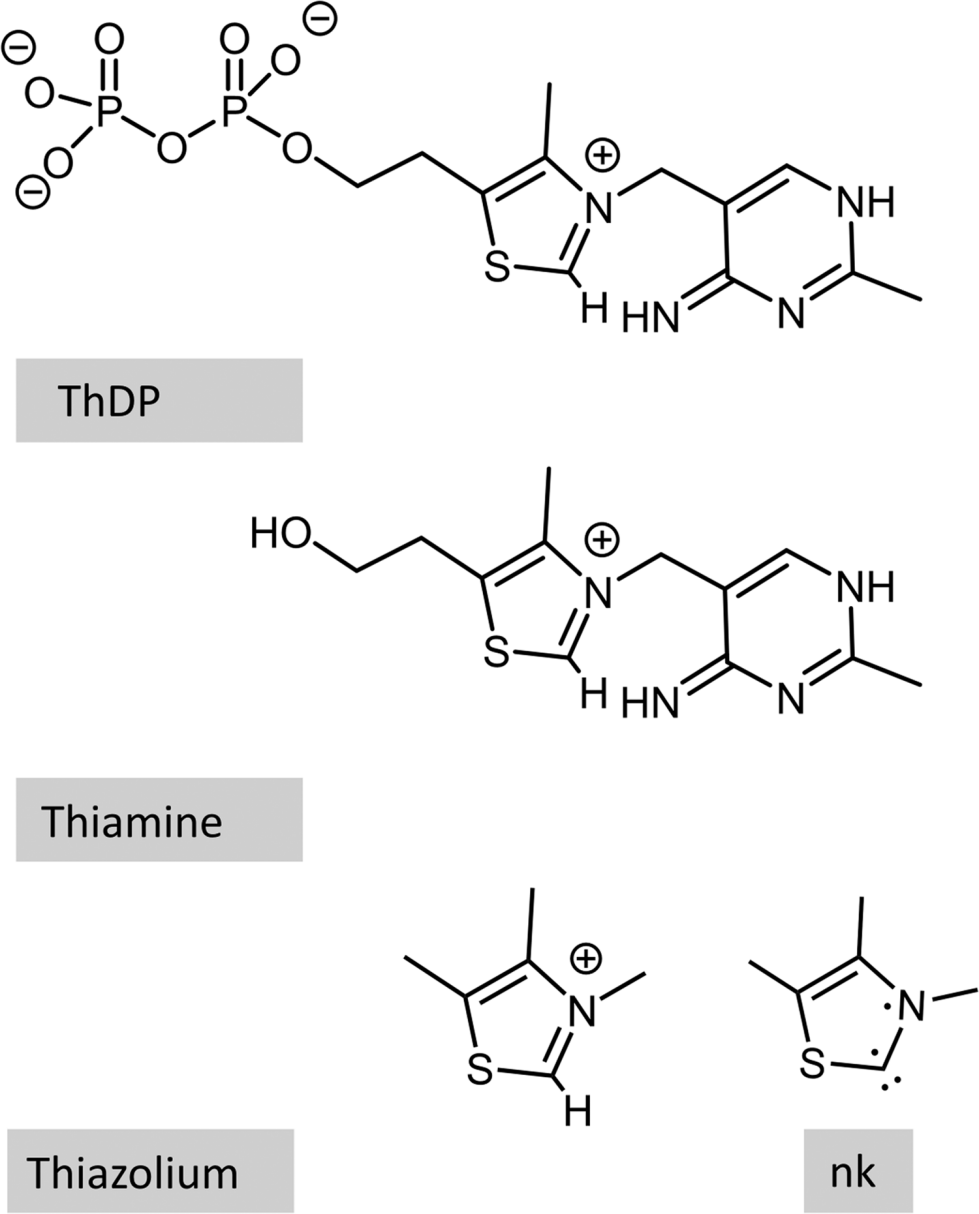
Lewis structures of ThDP cofactor, thiamine, and thiazolium molecules, showing the non-Kekule (nk) structure.

The strategy employed in our study has been to gradually increase the size of the system, to elucidate the origin of the p*K*_a_ shift (if present at all), see Figure 5, while at the same time comparing our results to previously reported literature values. First, we analyzed the thiazolium ring and the thiamine moieties. Then, we prepared cluster models in an effort to study the thiamine moiety along with some important residues that are found within the protein (see Figure 1).

### p*K*_a_ Values of Thiazolium and Thiamine

First, we present the results of the p*K*_a_ values obtained from QM calculations. All geometry optimizations and frequency calculations were performed with the Gaussian16 package,^21^ in the framework of density functional theory (DFT).^22,23^ Specifically we made use of the B3LYP^24,25^ functional together with Karlsruhe basis sets.^26,27^ Geometry and frequency calculations were done with the def2-SVPD basis set, employing the optimized geometries to perform single-point energy evaluations at def2-TZVPD with B3LYP and DSD-PBEP86 functionals.^28^ Harmonic vibrational frequencies were determined by analytical differentiation of gradients to determine the minima structure. The frequencies were then used to evaluate the Gibbs free energies. All of the calculations include Grimme’s empirical dispersion correction D3(BJ).^29,30^ In order to mimic the environment of the solvent, we have used the solvent model based on density (SMD) with the permittivity of water.^31^

The topology analysis was performed using the Multiwfn 3.8 software package.^32^ The Fuzzy partition scheme was employed to compute the bond orders,^33^ while Becke’s atomic dipole-corrected atomic charges were employed to analyze the atomic charges.^34^

#### Theoretical p*K*_a_ Values in Water

For the p*K*_a_ calculations, we consider the equilibria given by reaction 2.
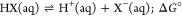
2

The p*K*_a_ values were obtained using the Gibbs free energy from geometry optimizations and frequency calculations. In order to obtain the absolute free energy of the proton in water, the free energy value of the proton in vacuum (−6.28 kcal/mol) and its solvation free energy (−265.63 kcal/mol) are employed.^35,36^

Theoretical p*K*_a_ estimates are often aided by the inclusion of two empirical parameters, as can be seen in eq 3 (*C*_0_ and *C*_1_).^37^ These two empirical parameters are said to correct different sources of errors.
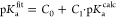
3

Recently, Thapa et al. have shown that the inclusion of explicit water molecules leads to very accurate theoretical p*K*_a_ estimates without the need of such empirical parameters. In other words, the empirical parameters foremost correct deficiencies in implicit solvation modeling.^38^

Herein, we use our previously parametrized model data in order to verify this observation, Table S1.^39^ Explicit water molecules were incorporated into the system by following the Lewis structure of the molecule being studied. These water molecules were specifically positioned to create hydrogen bonds with the polar hydrogen atoms and electron lone pairs. As a result, each polar hydrogen atom or electron lone pair is able to interact with an explicit water molecule.

The results show that indeed the addition of explicit water molecules leads to a better correlation coefficient, lower mean absolute deviation (MAD), and less need for empirical fitting: the slope comes close to unity, and the intercept gets slightly closer to zero (Figure S1 and Tables 1 and S1). Based on the obtained results, one can obtain accurate p*K*_a_ values in water environment either by applying the empirical corrections to the results which make use of a continuum solvation model or by explicitly including the solvent water molecules. It should also be noted that in the p*K*_a_ range of interest, our results exhibit a conservative error bar of about 4 units. One will see that the heteroscedastic estimate with microsolvation will be even smaller and robust enough for the values that we report on. To evaluate the performance of the method in the context of our current research, which focuses on the activation mechanism of ThDP leading to carbene formation, we have included three carbene molecules in Table S1. These molecules have known experimental values and serve as benchmarks to assess the accuracy and reliability of the introduced approach. As a final note, given the similar performance of the B3LYP and DSD-PBEP86 functionals, we will henceforth use the B3LYP functional for the remainder of this work.

**Table 1:** Summary of the Obtained Regression Parameters without (w/0) and with (w) Explicit Water Molecules for (a) B3LYP and (b) DSD-PBEP86^a^

	(a) w/0	(a) w	(b) w
*R*^2^	0.89	0.97	0.96
*C*_1_	0.67 ± 0.08	0.98 ± 0.06	0.95 ± 0.04
*C*_0_	0.82 ± 1.72	–0.74 ± 1.19	0.41 ± 0.71
MAD	1.99	0.88	0.85

a Mean absolute deviations (MAD) correspond to the differences obtained after the use of the empirical parameters.

#### Theoretical p*K*_a_ Values in Protein Environment

p*K*_a_ calculations for enzyme pockets are more complex, since explicit water molecules may be found at the surface but not necessarily in the buried regions. Moreover, the permittivity at the buried regions of the proteins is known to be variable, with hydrophobic pockets showing characteristically low values. In this work, we focus on the influence of neighboring residues. The influence of the permittivity is not within the scope of this work. Therefore, we employ the water permittivity value for all calculations, which allows us to compare to the reported experimental values, making use of the presented fitting. This enables us to compare relative p*K*_a_ differences and depict the effect of neighboring molecular groups.

#### Thiazolium in Solution

The documented p*K*_a_ value of the thiazoles-2-ylidenes is a characteristic attribute with a typical range between 17 and 19. Interestingly, it has been shown that this carbene species is able to form up to two hydrogen bonds.^16^ It has been postulated that these could offer up to 20 kcal/mol of stabilization.^13^ To verify whether such strong interactions lead to a possible shift of the p*K*_a_ value in the enzyme, we employ three different models: (1) without explicit water molecules, (2) with one explicit water molecule, and (3) with two explicit water molecules.

#### Thiamine in Solution

Then, the model is extended with the purpose of analyzing the p*K*_a_ values for the thiamine molecule in solution. In order to be pragmatic, an implicit solvent model is employed to represent the solvent. Note that in this case there are more titratable sites that together with the C_2_ position are studied herein. The goal is to systematically compare the p*K*_a_ values for the N_1′_, N_4′_, and C_2_ atoms, within the different tautomeric forms that can take place in the thiamine.

#### TKT Cluster Calculations

Finally, we extended the model, including some residues around the thiamine. The goal is to adequately represent the protein environment wrapping the thiamine cofactor, with the aim of analyzing their effect on the p*K*_a_ values. Thereby, we have truncated the X-ray crystal structure of the human TKT dimer (PDB code 4KXW) including some of the nearby residues to the ThDP.

We have selected the neighboring residues based on previous literature studies. In this sense, glutamate residues (E366, E160, and E165) were shown to be part of a proton-wire communication mechanism,^19^ the concomitant histidine residue (H110) was previously shown to be crucial for catalysis,^11^ and the water molecules were characterized through high-resolution X-ray structures,^3,19^ as important molecules in the catalytic event. Taking these points into consideration, we tried to construct a minimal cluster model that included these residues around the thiamine. The largest modeled system encompasses residues E366, E160, E165, and H110; two water molecules; and ThDP, whereas smaller models were constructed by omitting certain residues in order to assess their respective impacts. E366, E160, E165, and H110 are capped at the C_α_ atom, and ThDP was capped at the first oxygen binding the phosphate group, in order to simplify the model, which was substituted by an alcohol instead. All of the included residues were constrained at the C_α_ atom, and ThDP was constrained at the C atom of the alcohol-containing group and at N_1′_.

Given the presence of two explicit water molecules in the cluster model, we employ the linear regression correction with an explicit water molecule for this case.

**Figure 6:**
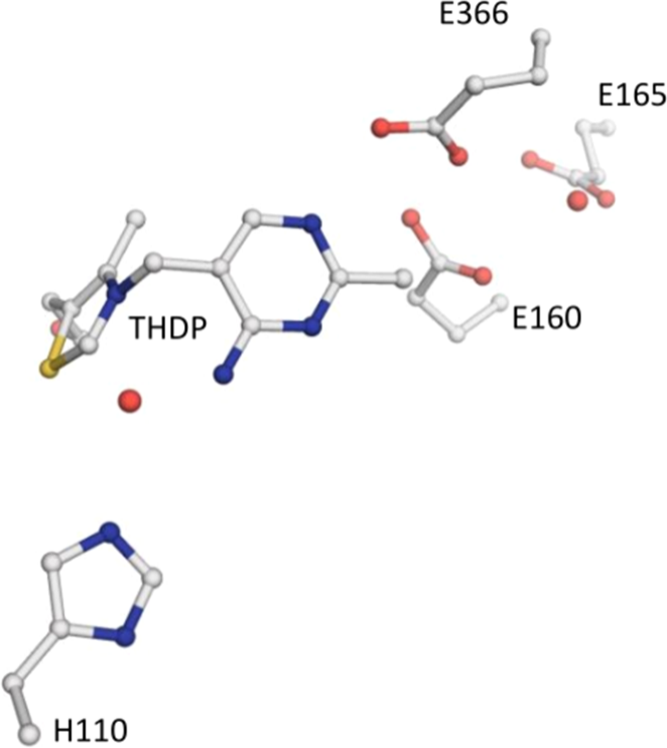
Representation of the cluster model residues, CM1 and CM2, from the original X-ray-resolved crystal structure.

First of all, the p*K*_a_ values of the histidine residue are analyzed, which is commonly known to be around 7. We have employed two different cluster models in order to analyze the effect of charge inclusion to the system. In this vein, the first cluster model (CM1) is formed by E366, E160, H110, and the cofactor, while a second cluster model (CM2) is also modeled adding E165 to the previous cluster; see Figure 6.

At this point, we compute the p*K*_a_ values for carbene formation employing various cluster models. We begin with a model comprised of thiamine, E366 and E160. We then expand the cluster system by first individually including E165 and H110, and finally, we include E165 and H110 simultaneously.

## Results and Discussion

### QM Calculations

#### Thiazolium in Solution

First, we evaluate the strength of the hydrogen bonds through the free energy of dissociation. The calculated interaction energy for the hydrogen bonds between carbene and the two water molecules is 13.2 kcal/mol, while the interaction with a single water molecule is about 7.9 kcal/mol. Taking the water dimer as a reference point, whose strength is measured to be 3.2 kcal/mol, these values show a stronger interaction compared to that of a conventional hydrogen bond.

As a sanity check, we compare our calculated *D*_0_ value with the experimentally measured one, for the dissociation of the water dimer (in gas phase), observing that the proposed methodology is able to reproduce this value.^40^

The obtained p*K*_a_ values are 17.7 ± 3.1 for the thiazolium without any explicit water molecules, 19.7 ± 1.4 with a single water molecule, and 18.2 ± 1.3 with two water molecules. It has to be noted that different *C*_0_ and *C*_1_ values are employed to obtain the mentioned p*K*_a_ values due to the lack or inclusion of explicit water molecules in each particular case. Overall, these results show that despite the observed strong hydrogen-bond interactions of the carbene with the water molecules its p*K*_a_ value is not lowered.

The structural information reveals that the S_1_–C_2_–N_3_ angle is shorter in the conjugate base, i.e., the carbene (106°) than in the acid, i.e., thiazolium, (112°) in line with structural observations.^3^ Moreover, a slight increase of the distances is also observed upon the formation of the carbene, 0.02 and 0.04 Å for C_2_–N_3_ and S_1_–C_2_ bonds, respectively, as it has already been reported.^3^

At last, we have explored the possibility of having a triplet (biradical) singlet carbene species. Taking the optimized carbene species and analyzing the molecular orbital energies, a very large HOMO–LUMO gap is observed. This already strongly hints against the suggested activation proposed by Hsu et al.^17^ Several optimizations were carried out for the isolated cofactor, in all three possible electronic states, starting from the singlet optimized geometry and from the distorted structure suggested by the authors. The obtained results show that the triplet state would be at least 60 kcal/mol higher than the closed-shell singlet species. Even if the enzyme environment could stabilize some of these distortions, it would be difficult to attain the intermediate. It is hard to stabilize a biradical with strong directed interactions, as this does not significantly change the charge distribution compared to the closed-shell state. It is more likely that the observed structural distortions are an artifact of the crystal structure data acquisition process.

#### Thiamine in Solution

**Figure 7:**
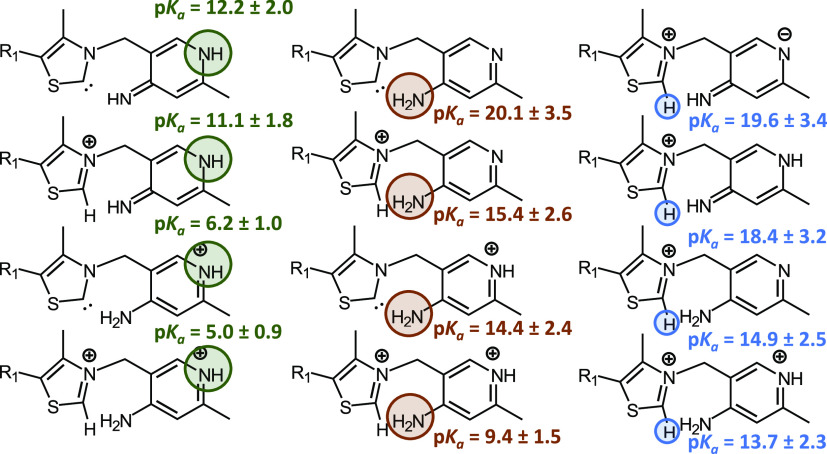
p*K*_a_ values of different tautomers of the thiamine molecule.

Depending on the protonation state of the N_4′_ atom, one can distinguish between aminopyrimidine (AP) and iminopyrimidine (IP) tautomers. It can be seen that the estimated p*K*_a_ values, for N_1′_ and C_2_, vary within these tautomeric states. Hence, the N_1′_ position shows a high p*K*_a_ value (11–12) in the IP tautomer. However, in the AP form of the thiamine, this p*K*_a_ is observed to decrease, about 6 units. In the same sense, the carbene formation is found to have a lower p*K*_a_ value when the AP form is found, where the interactions with the extra proton are found at the N_4′_ atom, see Tables S2 and S3. Finally, the protonation of the N_1′_ position slightly facilitates the formation of a carbene, lowering its p*K*_a_ value, for which the mentioned hydrogen-bond interaction is slightly stronger. All of the results are schematically presented in Figure 7.

The p*K*_a_ value for the carbene formation at the thiamine in solution has been reported to be 17.7 and 18.0 upon the protonated and deprotonated N_1′_, respectively.^14^ These reported values compare well to our computed values
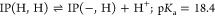
4
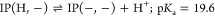
5which further solidifies the employed method and model. However, these values are far from the optimal pH of the enzyme. Previous works proposed either intramolecular (Reaction 6) or intermolecular (Reaction 7) thiamine activation pathways, departing from different reactant states
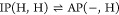
6
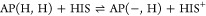
7which serves as further evidence of the lack of a general agreement. Employing the p*K*_a_ values obtained in this section, we estimated the energetic costs associated with the carbene formation. In order to estimate the intramolecular activation pathway, the whole reaction shown in reaction 6 can be written as the result of the subtraction (reactions 8 and 9) of the following reactions that were computed here
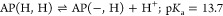
8
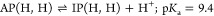
9

In a similar manner, employing reaction 8 and taking the reference p*K*_a_ value of histidine (7), we obtain an energy value associated with the intermolecular pathway. By doing so, both intramolecular and intermolecular formations of carbenes have been found to be endergonic processes, with free energy values of +5.87 and +9.14 kcal/mol, respectively. These values indicate a preference for the intramolecular activation mechanism. It is remarkable that this finding is in sharp contrast to previous theoretical studies which to our surprise, reported exergonic values for the formation of the unstable carbene species in an enzymatic environment.^9,10^

At this stage, it should be recalled that from our results the carbene formation exhibits a lowering of its p*K*_a_ value upon the presence of given tautomers (see Figure 7). Therefore, it is evident that the environment plays an important role in its formation. As the presence of suitable neighboring residues can effectively lower the p*K*_a_ value in the intermolecular mechanism, decreasing the energetic penalty and pulling the equilibrium toward the activated carbene species.

The same structural transitions as those for the thiazolium are observed in the case of the thiamine. We can observe a stretching of the C_2_–N_3_ and S_1_–C_2_ distances, together with a folding of the S_1_–C_2_–N_3_ angle upon the formation of the carbene species (see Table 2). Lastly, it has been previously noted that a mesomeric effect occurs in the thiamine moiety,^10^ which is said to be the cause of the favorable energetic observed for the carbene formation. This is determined by the measured short distance between the amino N_4′_ atom and the C_4′_ atom of the pyrimidine ring (C_4′_–N_4′_ bond). The distances in question are presented in Table 2, and it can be observed that the C_4′_–N_4′_ distance is indeed short and comparable to previously reported values. However, even if mesomerism is also present in our results, it is clear that it does not justify favorable carbene formation at near-neutral pH values.

**Table 2:** Thiamine Distances and Angles in the Thiazolium Moiety (B3LYP-D3(BJ)/def2-SVPD)

	C_2_–N_3_ (Å)	S_1_–C_2_ (Å)	S_1_–C_2_–N_3_ (deg)	C_4′_–N_4′_ (Å)
AP(−,−)	1.34	1.72	106.4	1.35
AP(H,−)	1.32	1.69	112.3	1.35
AP(−,H)	1.34	1.72	106.4	1.33
AP(H,H)	1.33	1.69	112.2	1.33
IP(−,−)	1.34	1.72	106.4	1.32
IP(H,−)	1.32	1.70	112.2	1.32
IP(−,H)	1.34	1.72	106.3	1.30
IP(H,H)	1.32	1.69	112.1	1.30

#### Reaction Mechanism

The results gathered until this point show the characterization of the protonation state of thiamine in solution is already a highly intricate issue, as different tautomeric forms can coexist. Therefore, we would like to take the opportunity to analyze the effect of different protonation states on the reaction barrier for the attack of the carbene to the carbonyl moiety of substrate X5P.

**Figure 8:**
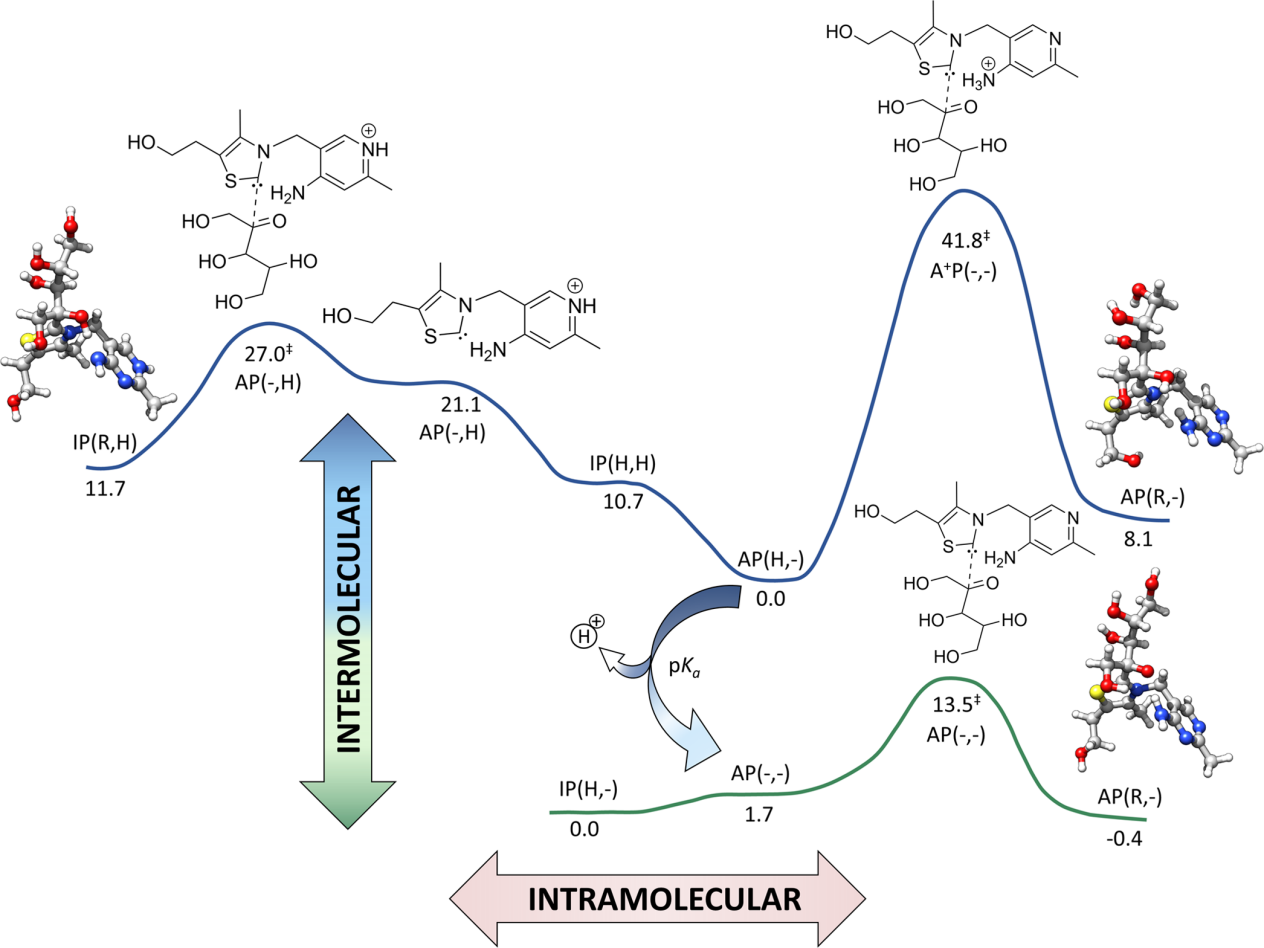
Computed reaction pathway for the attack thiamine intermolecular and intramolecular activation of the carbene with the subsequent attack at the carbonyl moiety of X5P. Free energies are shown in kcal/mol.

The results in Figure 8 show that the barrier is considerably diminished (about 10–30 kcal/mol) upon the loss of a proton. Moreover, it can be seen that the carbene species is almost isoenergetic (+1.7 kcal/mol) to the IP tautomer, making this activated species accessible; again, upon the loss of a proton. On the other hand, the presence of an added proton breaks this equilibrium and the carbene species becomes energetically not as accessible (+21.1 kcal/mol).

#### Cluster Calculations

The carbene species has been shown to have the lowest energetic demand if formed from the AP form. Its p*K*_a_ value has already been presented to be around 14–15. However, the optimal pH of the enzyme is known to be in the range of 7.5–8.6, which makes this process endergonic with Δ*G*^0^ = ln(10)*RT*(p*K*_a_ – pH). In this vein, neighboring residues should play an important role,^11^ lowering the p*K*_a_ value for the activation of ThDP. Motivated by this issue, herein, we present results within the cluster approach, which has previously been shown to be a useful method for computing reaction mechanisms.^41^

Specifically, we focus on residue H110, which has been shown to be crucial in the catalytic event. We analyzed the p*K*_a_ value of this residue which is observed to vary upon the presence of different tautomeric forms of the cofactor, see Table 3. It is interesting to note that in the presence of a positive charge at the thiamine, the p*K*_a_ of H110 drops to 4.5. On the other hand, the value increases in the presence of the carbene species. It is also interesting to note that the presence of the negatively charged E165 systematically increases the p*K*_a_ of H110, further stabilizing the positive charge.

**Table 3:** QM Computed p*K*_a_ Values of H110 in TKT for the AP(H,H)-, IP(H,H)-, and AP(−,H)-Containing Tautomers

	QM (CM1)	QM (CM2)
AP(H,H)	4.5 ± 0.5	4.7 ± 0.5
AP(−,H)	8.4 ± 0.6	9.6 ± 0.7
IP(H,H)	7.3 ± 0.6	8.6 ± 0.6

**Figure 9:**
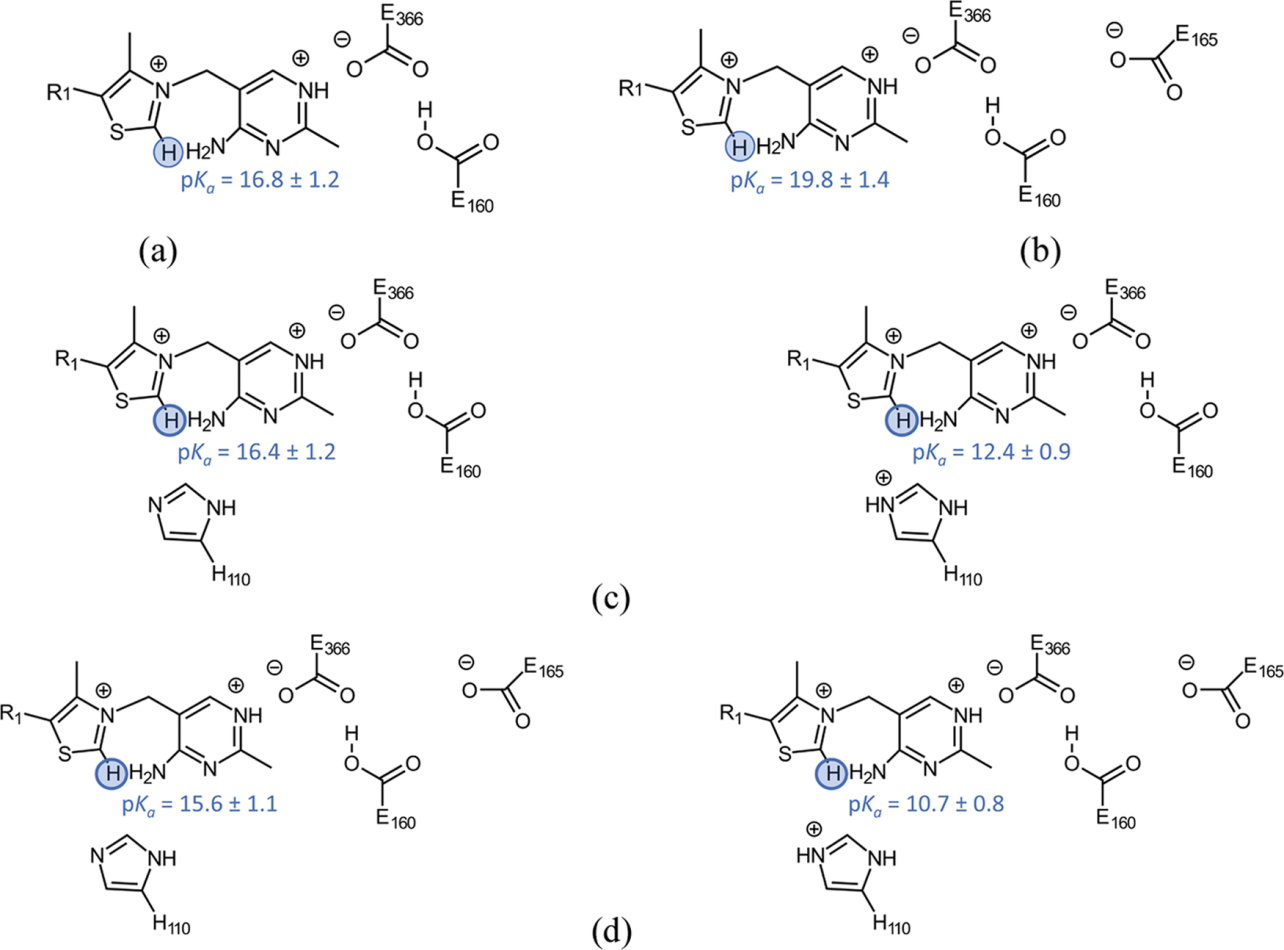
p*K*_a_ values of thiamine C_2_ in the presence of different protonation states of H110. Four different cluster models are shown, which include residues: (a) E366 and E160, (b) E366, E160, and E165, (c) E366, E160, and H110, and (d) E366, E160, E165, and H110.

Based on the obtained p*K*_a_ shifts for H110 upon the presence of a positive charge, we have explored this same possibility for the activation mechanism of ThDP. We have previously shown that the formation of the carbenes has characteristically high p*K*_a_ values, around 18 for the thiazolium and the IP tautomer of the thiamine and around 15 for the AP tautomer. Now, we have varied the protonation state of H110, analyzing its influence on the activation of thiamine. From the obtained values, Figure 9, it is visible that upon the protonation of H110, the p*K*_a_ for the carbene formation drops considerably to a value of 10.7. In this vein, if residue H110 is deprotonated or removed, the p*K*_a_ value of the carbene increases again to its regular value (16–20). Therefore, the results show that H110 is able to modulate the activation of ThDP cofactor by providing an adequate environment for the formation of a carbene species at a surprisingly low pH. The obtained p*K*_a_ value is close to the optimal pH of the enzyme, and so in the presented scenario, the formation of the carbene would be close to equilibrium.

These results indicate a distinctive pattern wherein the accumulation of positively charged residues facilitates the carbene formation by decreasing the p*K*_a_ value for its formation. However, these results should be considered with caution, as the exact modeling of the active site through further inclusion of residues is challenging. The main challenge stems from the slow convergence of electrostatic interactions with respect to the cutoff radius in relation to charged residues.^42^ Despite our efforts to include the most relevant residues, limitations of the cluster model must be acknowledged. The TKT protein in complex with ThDP possesses a multitude of charged residues in the vicinity of the thiamine moiety, which may significantly impact this p*K*_a_ value. One can observe that the calculated p*K*_a_ value remains relatively constant for the AP(H,H) system (varying only by 0.2 p*K*_a_ units), whereas the inclusion of E165 has a more significant effect on the IP(H,H) and AP(−,H) systems, resulting in fluctuations of about 1 p*K*_a_ unit. The reason for this deviation can be attributed to the overall charge of the cluster model. Specifically, the AP(H,H) system begins with a positive cluster that, upon the loss of a proton, results in a different positive or neutral charge of the system. In contrast, the IP(H,H) and AP(−,H) systems start in a neutral or positive state but end with a negative or neutral overall charge of the system, leading to higher p*K*_a_ value fluctuation.

## Conclusions

In the present work, we investigated the activation mechanism of ThDP in the TKT system. Adopting a bottom-up strategy, we used quantum mechanical (QM) calculations to study the p*K*_a_ values of the C2 atom in thiazolium and thiamine, both in an aqueous environment. In addition, we included previously characterized key enzyme residues (H110, E366, E160, and E165) and gradually increased the system size for a more complete analysis that includes the effect of the protein environment.

Overall, we conclude that the activation of ThDP is mediated by a p*K*_a_ shift modulated by the adjacent H110 residue, which has been shown to be critical for catalytic activity.^11^ In this sense, our cluster calculations show that the protonated form of H110 significantly lowers the p*K*_a_ value of C_2_ (about 7 pH units), making the carbene species accessible at the observed optimal pH of the enzyme (pH around 8). In the calculated reaction mechanisms, the proposed activation by acid–base equilibrium was also shown to lead to a lowering of the free energy barrier, consistent with the experimentally observed k_*cat*_. As a result, this study provides a solid foundation that paves the way for exploring the intricate catalytic activity of ThDP. However, it is important to note that the cluster calculations performed in this study included only a few residues deemed to play a pivotal role in TKT. Consequently, one would gain from further studies including the enzyme environment (QM/MM) by building upon the current data.

In a previous work,^19^ we had already highlighted how carbene activation can be steered by neighboring hydrogen-bonding networks. This provides a (to date) unique allosteric mechanism, although we expect further examples to come to light in the near future. The difficulty in capturing proton dynamics makes it particularly hard to capture such activation processes, a task made even more challenging by the difficulty in modeling protonation changes with classical force fields. The results here obtained are able to clear some of the questions left open by other proposed mechanisms and set the stage for further experimental work on ThDP-dependent enzymes. In particular, how can one potentially model the reactivity by the design of local electrostatic fields close to the cofactor.

## Data Availability

The data generated in this study is available at 10.25625/ZWKPMD.

## Supporting Information Available

p*K*_a_ values for both the experimental and computed molecules (Table S1); topology analyses indicating the bond orders and atomic charges of thiamine in solution (Tables S2 and S3); linear regressions of the p*K*_a_ estimations without and with explicit water molecules (Figure S1) (PDF)
