# Supplementary material for: Dynamic Protonation States Underlie Carbene Formation in ThDP-Dependent Enzymes: A Theoretical Study
Source: J Phys Chem B. 2023 Sep 25;127(44):9423–32. doi: 10.1021/acs.jpcb.3c03137 (PMC10688766; doi:10.1021/acs.jpcb.3c03137)
Supplement: Supplementary file 1 — jp3c03137_si_001.pdf [file jp3c03137_si_001.pdf]

Supporting Information: Dynamic Protonation States underlie Carbene Formation in ThDP-Dependent Enzymes: A Theoretical Study

Jon Uranga,*,† Fabian Rabe von Pappenheim,‡ Kai Tittmann,‡,¶ and Ricardo A. Mata*,†

†*Institute of Physical Chemistry, Georg-August Universität Göttingen, Tammannstraße 6, 37077, Göttingen, Germany*

‡*Department of Molecular Enzymology, Göttingen Center of Molecular Biosciences, Georg-August Universität Göttingen, Julia-Lermonotowa-Weg 3, D-37077 Göttingen, Germany*

¶*Department of Physical Biochemistry, Max-Planck-Institute for Multidisciplinary Natural Sciences, Am Faßberg 11, D-37077 Göttingen, Germany*

E-mail: juranga@gwdg.de; rmata@gwdg.de

Table S1: Studied molecules' experimental and theoretical acidities without (w/0) and with (w) explicit water molecules. (a) 4-(hydroxythio)-1-methylpyrimidin-2(1H)-one, (b) 1,3-diethyl-3,4,5,6-tetrahydropyrimidin-1-ium, (c) 3-ethyl-1methyl-1H-imidizaol-3-ium and (d) 1,3-bis(4-methoxyphenyl)-1H-imidazol-3-ium. The number of explicit water molecules is indicated in parenthesis.

Molecule	$pK_a{}^{exp}$	$pK_a{}^{B3LYP}$ (w/0)	$pK_a{}^{B3LYP}$ (w)	$pK_a{}^{DSD-PBEP86}$ (w)
Acetic	4.8[1]	8.01	5.14 (4)	4.30 (4)
Formic	3.8[1]	5.52	2.81 (4)	2.17 (4)
Orotic	0.4[2]	1.97	2.31 (4)	1.37 (4)
Benzoic	4.2[1]	7.28	5.38 (4)	4.18 (4)
Methanol	15.5[1]	25.87	15.35 (3)	15.08 (3)
Ethanol	15.9[1]	26.35	17.15 (3)	16.74 (3)
Phenol	10.0[1]	15.69	10.05 (3)	9.25 (3)
Ammonium	9.25[3]	8.46	11.0 (4)	10.41 (4)
Pyridinium	5.3[1]	6.21	6.60 (1)	5.09 (1)
Imidazolium	7.0[1]	7.37	7.76 (2)	6.25 (2)
Pyrimidinium	1.3[1]	1.67	2.22 (2)	0.68 (2)
Anilinium	4.6[1]	1.96	5.87 (3)	4.83 (3)
Guanidinium	13.8[1]	17.04	15.73 (6)	14.48 (6)
Hydrogen Peroxide	11.7[4]	18.39	12.26 (6)	11.96 (6)
Methanethiol	10.4[5]	21.87	10.34 (3)	9.42 (3)
Methylperoxide	11.5[6]	18.79	16.78 (5)	16.84 (5)
(a)	6.3[7]	8.62	7.57 (3)	6.11 (3)
Trimethylamine	10.65[8]	10.69	11.15 (1)	10.54 (1)
(b)	27.8[9]	34.64	27.94 (2)	27.07 (2)
(c)	23.0[9]	29.30	22.91 (2)	21.90 (2)
(d)	20.7[9]	24.88	22.65 (2)	21.12 (2)

Table S2: Thiamine Fuzzy bond orders in the thiazolium and pyrimidine moieties (B3LP-D3(BJ)/def2-TZVPD).

	AP(-,-)	AP(H,-)	AP(-,H)	AP(H,H)	IP(-,-)	IP(H,-)	IP(-,H)	IP(H,H)
S_1-C_2	1.50	1.44	1.50	1.44	1.51	1.42	1.51	1.43
C_2-N_3	1.48	1.46	1.47	1.46	1.49	1.46	1.49	1.46
N_3-C_4	1.18	1.21	1.18	1.20	1.18	1.20	1.18	1.20
C_4-C_5	1.50	1.46	1.50	1.46	1.50	1.47	1.50	1.47
C_5-S_1	1.25	1.27	1.25	1.27	1.25	1.26	1.25	1.27
$N_{1'}$-$C_{2'}$	1.50	1.48	1.35	1.35	1.47	1.46	1.32	1.31
$C_{2'}$-$N_{3'}$	1.50	1.52	1.59	1.58	1.58	1.58	1.68	1.68
$N_{3'}$-$C_{4'}$	1.44	1.42	1.37	1.38	1.33	1.33	1.27	1.27
$C_{4'}$-$C_{5'}$	1.22	1.24	1.18	1.51	1.15	1.15	1.11	1.11
$C_{5'}$-$C_{6'}$	1.45	1.42	1.52	1.50	1.50	1.49	1.58	1.57
$C_{6'}$-$N_{1'}$	1.50	1.53	1.37	1.38	1.49	1.50	1.33	1.34
$C_{4'}$-$N_{4'}$	1.44	1.43	1.52	1.51	1.72	1.71	1.79	1.79
C_2- - -H	0.08	0.01	0.10	0.01	-	-	-	-

Table S3: Becke's dipole moment corrected atomic charges of Thiamine in solution.

	AP(-,-)	AP(H,-)	AP(-,H)	AP(H,H)	IP(-,-)	IP(H,-)	IP(-,H)	IP(H,H)
S_1	0.09	0.19	0.10	0.22	-0.17	0.10	-0.09	0.12
C_2	-0.50	-0.21	-0.48	-0.21	-0.15	-0.09	-0.32	-0.21
N_3	0.12	0.11	0.11	0.06	-0.21	0.32	-0.09	0.47
C_4	0.05	0.08	0.05	0.09	0.41	0.02	0.20	0.00
C_5	-0.02	-0.01	-0.02	-0.01	-0.15	-0.06	0.02	-0.04
$N_{1'}$	-0.46	-0.58	-0.10	-0.26	-0.77	-0.87	-0.36	-0.50
$C_{2'}$	0.21	0.39	0.17	0.25	0.48	0.48	0.32	0.37
$N_{3'}$	-0.38	-0.37	-0.32	-0.32	-0.42	-0.52	-0.37	-0.35
$C_{4'}$	0.14	0.12	0.20	0.33	0.10	0.11	0.21	0.18
$N_{4'}$	-0.53	-0.73	-0.35	-0.89	-0.79	-1.28	-0.67	-0.60
$C_{5'}$	0.08	0.06	-0.03	-0.06	0.27	0.11	0.05	-0.04
$C_{6'}$	0.34	0.06	0.05	0.10	-0.26	0.22	-0.12	0.13

Figure S1: Linear regressions of the studied molecules at B3LYP level a) without and b) with water molecules.

(a)

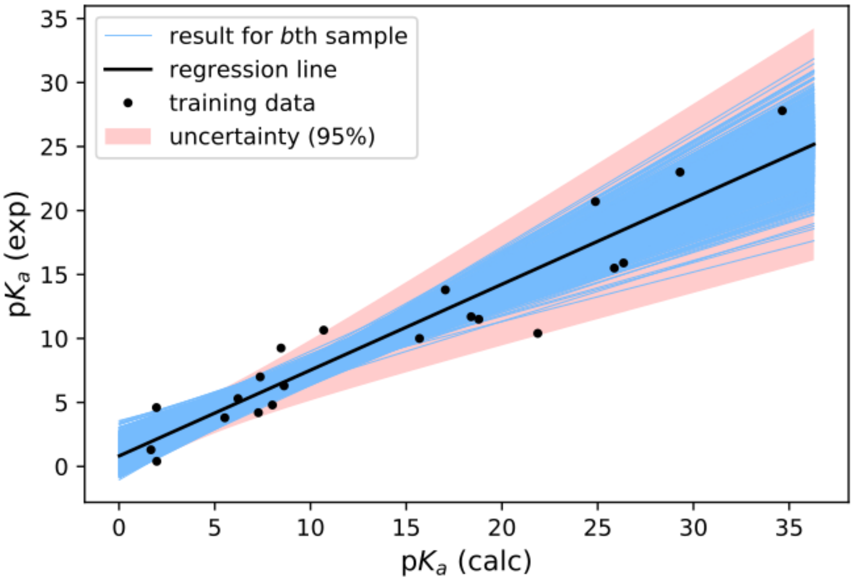

(b)

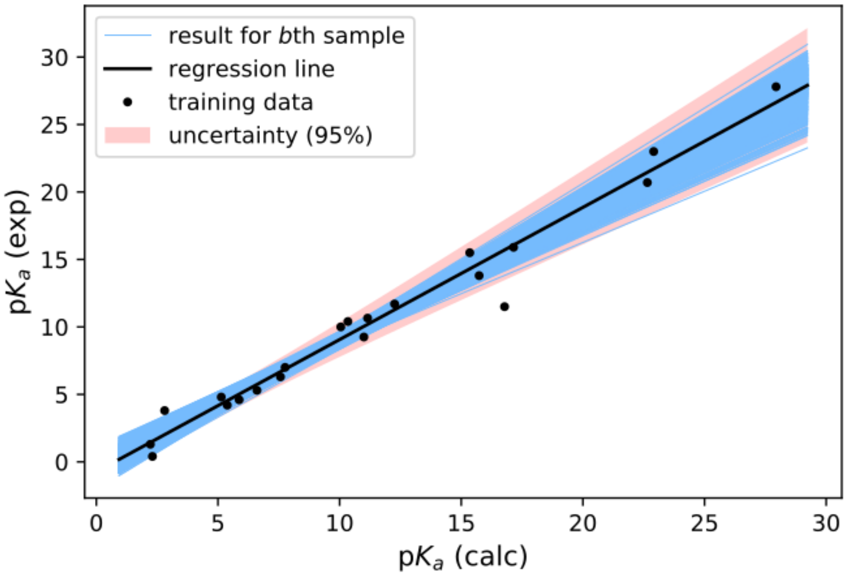

References

(1) Klicić, J. J.; Friesner, R. A.; Liu, S. Y.; Guida, W. C. Accurate Prediction of Acidity Constants in Aqueous Solution via Density Functional Theory and Self-Consistent Reaction Field Methods. *J. Phys. Chem. A* **2002**, *106*, 1327–1335.

(2) Radzicka, A.; Wolfenden, R. A Proficient Enzyme. *Science* **1995**, *267*, 90–93.

(3) Ho, J.; Coote, M. L. A Universal Approach for Continuum Solvent pKa Calculations: Are We There Yet? *Theor. Chem. Acc.* **2010**, *125*, 3–21.

(4) Charron, I.; Couvert, A.; Laplanche, A.; Renner, C.; Patria, L.; Requieme, B. Treatment of Odorous Sulphur Compounds by Chemical Scrubbing with Hydrogen Peroxide - Stabilisation of the Scrubbing Solution. *Environ. Sci. Technol.* **2006**, *40*, 7881–7885.

(5) El-Sayed, A.; Shindia, A. Characterization and Immobilization of Purified Aspergillus Flavipesl-Methioninase: Continuous Production of Methanethiol. *J. Appl. Microbiol.* **2011**, *111*, 54–69.

(6) Richardson, W.; Hodge, V. Acidities of Tertiary Alkyl Hydroperoxides. *J. Org. Chem.* **1970**, *35*, 4012–4016.

(7) Gupta, V.; Carroll, K. S. Sulfenic Acid Chemistry, Detection and Cellular Lifetime. *BBA-Gen. Subjects* **2014**, *1840*, 847 – 875.

(8) Hollóczki, O. The Mechanism of N-Heterocyclic Carbene Organocatalysis through a Magnifying Glass. *Chem–Eur. J.* **2020**, *26*, 4885–4894.

(9) Higgins, E. M.; Higgins, E. M.; Armstrong, J.; Massey, R. S.; Alder, R. W.; O'Donoghue, A. M. C. pK as of the Conjugate Acids of N-Heterocyclic Carbenes in Water. *Chem. Commun.* **2011**, *47*, 1559–1561.
